# Clinical Implementation of Integrated Genomic Profiling in Patients with Advanced Cancers

**DOI:** 10.1038/s41598-016-0021-4

**Published:** 2016-12-23

**Authors:** Mitesh J. Borad, Jan B. Egan, Rachel M. Condjella, Winnie S. Liang, Rafael Fonseca, Nicole R. Ritacca, Ann E. McCullough, Michael T. Barrett, Katherine S. Hunt, Mia D. Champion, Maitray D. Patel, Scott W. Young, Alvin C. Silva, Thai H. Ho, Thorvardur R. Halfdanarson, Robert R. McWilliams, Konstantinos N. Lazaridis, Ramesh K. Ramanathan, Angela Baker, Jessica Aldrich, Ahmet Kurdoglu, Tyler Izatt, Alexis Christoforides, Irene Cherni, Sara Nasser, Rebecca Reiman, Lori Cuyugan, Jacquelyn McDonald, Jonathan Adkins, Stephen D. Mastrian, Riccardo Valdez, Dawn E. Jaroszewski, Daniel D. Von Hoff, David W. Craig, A. Keith Stewart, John D. Carpten, Alan H. Bryce

**Affiliations:** 1 Division of Hematology/Oncology Mayo Clinic, Scottsdale, AZ USA; 2 Mayo Clinic Cancer Center, Scottsdale, AZ USA; 3 Center for Individualized Medicine, Mayo Clinic, Rochester, MN USA; 4 Translational Genomics Research Institute, Phoenix, AZ USA; 5 Department of Pathology, Mayo Clinic, Scottsdale, AZ USA; 6 Department of Biomedical Statistics and Informatics, Mayo Clinic, Scottsdale, AZ USA; 7 Department of Radiology, Mayo Clinic, Scottsdale, AZ USA; 8 Mayo Clinic Cancer Center, Rochester, MN USA; 9 Department of Cardiovascular Surgery, Mayo Clinic, Scottsdale, AZ USA

## Abstract

DNA focused panel sequencing has been rapidly adopted to assess therapeutic targets in advanced/refractory cancer. Integrated Genomic Profiling (IGP) utilising DNA/RNA with tumour/normal comparisons in a Clinical Laboratory Improvement Amendments (CLIA) compliant setting enables a single assay to provide: therapeutic target prioritisation, novel target discovery/application and comprehensive germline assessment. A prospective study in 35 advanced/refractory cancer patients was conducted using CLIA-compliant IGP. Feasibility was assessed by estimating time to results (TTR), prioritising/assigning putative therapeutic targets, assessing drug access, ascertaining germline alterations, and assessing patient preferences/perspectives on data use/reporting. Therapeutic targets were identified using biointelligence/pathway analyses and interpreted by a Genomic Tumour Board. Seventy-five percent of cases harboured 1–3 therapeutically targetable mutations/case (median 79 mutations of potential functional significance/case). Median time to CLIA-validated results was 116 days with CLIA-validation of targets achieved in 21/22 patients. IGP directed treatment was instituted in 13 patients utilising on/off label FDA approved drugs (n = 9), clinical trials (n = 3) and single patient IND (n = 1). Preliminary clinical efficacy was noted in five patients (two partial response, three stable disease). Although barriers to broader application exist, including the need for wider availability of therapies, IGP in a CLIA-framework is feasible and valuable in selection/prioritisation of anti-cancer therapeutic targets.

## Introduction

The advent of next-generation sequencing (NGS) has enabled high-throughput, real-time interrogation of cancer genomes. While initial efforts focused on large scale mapping of cancer genomes to elucidate disease pathogenesis, novel therapeutic targets and prognostic marker discovery^1^, more recent efforts have also considered the application of NGS to individual patients^2–5^.

In a study conducted by Von Hoff and colleagues, molecular profiling focusing on clinically available therapies was applied to the care of patients with advanced, refractory cancers^6^. Using a panel of 11 proteins assayed by IHC and FISH, and 51 genes using oligonucleotide microarrays, 84 of 86 (98%) of patients were found to have a putatively actionable therapeutic target. Sixty-six of the 84 patients were treated with therapy directed by molecular profiling. Eighteen of 66 treated patients (27%) were found to have progression-free survival (PFS) ≥1.3 times longer on the molecular profiling directed approach compared to their most recent empiric therapy (PFS ratio), allowing for rejection of the null hypothesis of <15% having PFS ratio ≥1.3.

In a similar effort focused on patients treated on Phase I trials, Tsimberidou and colleagues assessed microdissected paraffin embedded tissue for somatic hot-spot exonal mutations in 10 genes (*PIK3CA*, *BRAF*, *NRAS*, *KRAS*, *EGFR*, *KIT*, *GNAQ*, *MET*, *TP53* and *RET*), IHC for PTEN and FISH for *ALK* translocations in 1,144 patients^7^. They found that 460 (40.2%) of these patients had at least one genomic aberration. Notably, a higher response rate (27% vs. 5%; *P* < 0.0001), time-to-treatment failure (median 5.2 vs. 2.2 months; *P* < 0.0001) and longer survival (median 13.4 vs. 9.0 months; *P* = 0.017) were observed in patients who had matched therapy compared to those patients who had empiric therapy.

While the scope of molecular profiling performed in these studies was limited compared to current NGS enabled approaches, they provide a contextual platform upon which clinical efforts utilising NGS could be implemented. NGS based approaches assaying a panel of genes^4^ or a panel of genes in conjunction with array comparative genomic hybridisation (aCGH)^8^, provided further impetus towards more ambitious, comprehensive genomic characterisation in the clinical setting. More recently, a retrospective study by Jones and colleagues comparing whole exome sequencing to NGS panel based approaches, highlighted the high false positive rate and absence of germline analysis associated with NGS panels as major limitations^9^. Several other recent studies have further demonstrated the necessity of including germline analysis when evaluating somatic mutations^10–13^.

Exploratory evaluations of whole exome, genome and transcriptome sequencing have demonstrated the technical feasibility of the approach. However, these efforts were limited (Weiss and colleagues [n = 9]^3^ and Roychowdhury and colleagues [n = 4]^5^) and identified numerous barriers that should be evaluated in future studies. These barriers include the ability to execute the workflow consistently in a Clinical Laboratory Improvement Amendments (CLIA)^14^ environment and challenges in acquisition of sufficient, high-quality, NGS suitable tissue from prospectively collected samples. Furthermore, ethical, legal, and social implications (ELSI) are significant as these encompass the communication of incidental (unsolicited) findings from germline analysis, data custody and data privacy in the event of death prior to result availability. In addition, the delivery of results to treating physicians in a timeframe that is compatible with the opportunity to treat patients experiencing clinical decline while awaiting results and access to genome analysis guided therapeutics through on/off-label use of drugs or clinical studies, present very real barriers to implementation of comprehensive NGS technologies in the clinic.

In response to these observed challenges, a study was designed to evaluate the effectiveness of implementation of comprehensive NGS technologies in a clinical setting. The study objectives were threefold. First, the study sought to estimate the time to completion of integrated whole exome/long insert whole genome/transcriptome sequencing. Second, the objective was to estimate time to reporting of results of therapeutically relevant drug targets derived from integrated whole exome/long-insert whole genome/whole transcriptome sequencing along with CLIA validation. Finally, the study sought to determine mechanisms of drug access.

## Results

### Enrollment & Tissue Acquisition

From July 2010 to March 2013, 64 patients (n = 19 pilot phase; n = 45 CLIA phase) with advanced cancer provided signed, informed consent to this study using two institutional review board (IRB) approved protocols for advanced cancers. Overall, 35 patients, (54.6% of those consented), 6 in the pilot and 29 in the CLIA phase, were enrolled into the study and proceeded with tissue acquisition and molecular analysis of their tumours. The majority of the enrolled patients presented to the study with an Eastern Cooperative Oncology Group Performance Status (ECOG PS) of 1 (Table 1). Of note, five of these patients required a repeat tissue acquisition procedure in order to obtain sufficient tissue to complete the molecular analyses.

**Table 1:** Demographics and pathological characteristics.

Age Range (median years)		27–91 (59)
Sex, % (n)	Male	62% (22)
	Female	38% (13)
Ethnicity, % (n)	White	97% (34)
	Asian	3% (1)
Number of Biopsies to Acquire Sufficient Quality Tissue, % (n)^a^	1 Biopsy	88% (30)
	2 Biopsies	12% (5)
Biopsy Type, % (n)	Core	51.4% (18)
	VATS^b^	20% (7)
	Excision	14.3% (5)
	Other^c^	14.3% (5)
Tumour Type, % (n)	Pancreatic Cancer	28% (10)
	Cholangiocarcinoma	20% (7)
	Multiple Myeloma	6% (2)
	Hepatocellular Carcinoma	6% (2)
	Other^d^	40% (14)
ECOG Performance Status, % (n)	ECOG 1	94% (33)
	ECOG 2	3% (1)
	ECOG 3	3% (1)
Prior Cancer Treatments, Range (median)		0–6 (1)
Prior Radiation Treatments, % (n)	Yes	17% (6)
	No	83% (29)
Prior Surgery, % (n)	Yes	37% (13)
	No	63% (22)
Malignant Tumour Cellularity Range (median)^e^		2–100% (53%)
Benign Tumour Cellularity Range (median)^e^		0–98% (40%)
Percent Necrosis Range (median)^e^		0–60% (9.8%)

^a^For one patient, sufficient tissue for analysis could not be obtained.

^b^Video-assisted thoracic surgery.

^c^One each: laparatomy , esophagogastroduodenoscopy (EGD), debulking, tonsillectomy, bronchoscopy.

^d^One each: liposarcoma, stomach cancer, oropharynx cancer, gastroesophageal cancer, cervical cancer, lung adenocarcinoma, gallbladder cancer, bladder cancer, basal cell carcinoma, melanoma, mesothelioma, testicular cancer, uterine cancer, renal cell carcinoma.

^e^Calculated based on the average of all specimens collected for each individual patient.

**Figure 1:**
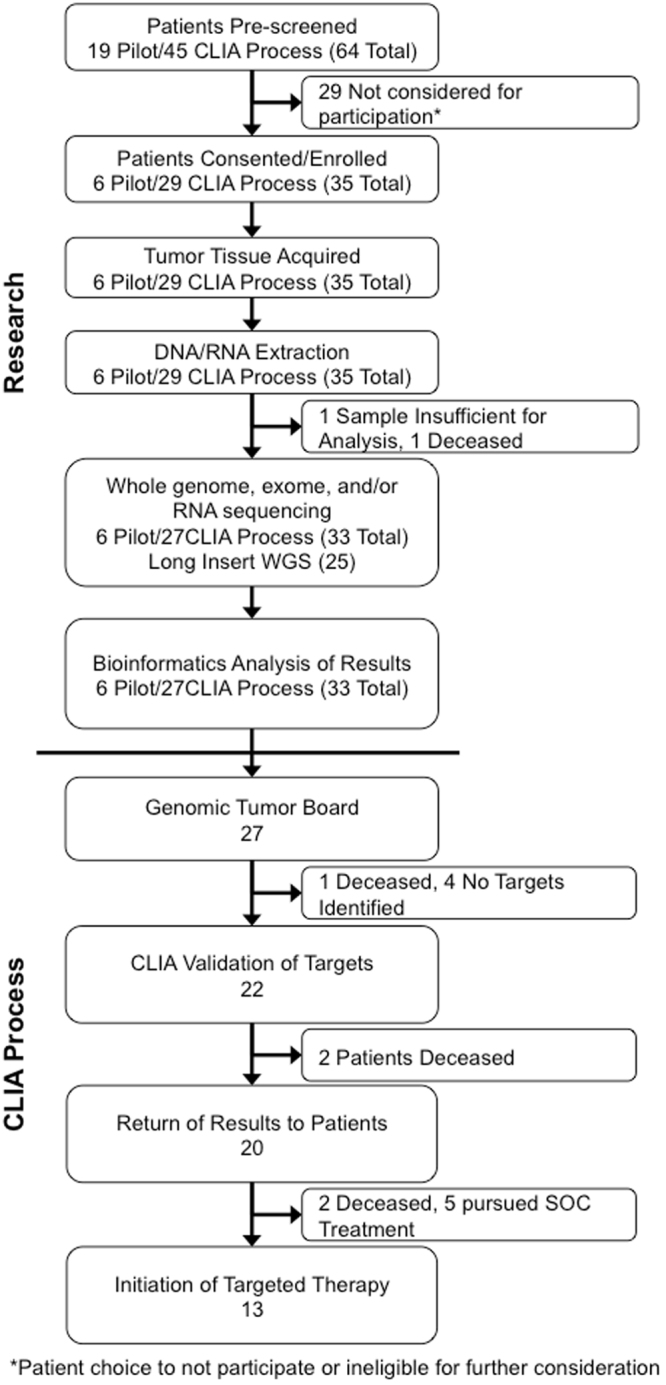
CONSORT Diagram of the sequencing workflow used in this pilot study including the number of patients at each step.

Figure 1 outlines the research and CLIA validation processes that were followed for this pilot study, noting that the initial phase of the study was completed entirely under the research platform. Those patients enrolled into the CLIA phase of the study had their results confirmed under the purview of CLIA validation for clinical implementation of profiling-directed targeted therapy. As indicated in Fig. 1, 64 patients were pre-screened, but ultimately 29 were not considered for participation either due to patient choice not to continue or failure to meet eligibility criteria. As a result, 35 patients (6 in the pilot and 29 in the CLIA phase) enrolled in the study. Sixteen of these 29 CLIA-phase patients did not complete the entire process for a multitude of reasons including: insufficient isolation of DNA/RNA, death, absence of actionable targets, and the decision to pursue treatment options available through routine clinical use. Clinically available/routine therapy not directed by profiling was implemented due to inability to access therapy, continued success without progression while on the standard of care regimen or treating physician discretion given clinical context supported by contraindications, comorbidities and patient-specific factors. In those instances where targeted therapy was pursued (n = 13), 9 patients received an off label FDA approved cancer drug, three of them enrolled in a clinical trial, one accessed drug through single patient IND and one was unable to access the recommended drug (Table 2).

**Table 2:** Summary of identified targets, treatments and responses.

	Patient	Tumor type	Actionable targets	Drug Category	Level of Evidence	Best response*	Treatment access method
**Empirical/ Non-target directed therapy (n = 5)**	1	Cholangiocarcinoma	**NRAS G13R, IDH2 R42W**	Cytotoxic	3	NA	Routine clinical mechanisms
	2	Gastroesophageal cancer	**NRG1 I711M, ABL1 P801R, ERBB2 RNA log2 = 4.48**	Cytotoxic	1	NA	Routine clinical mechanisms
	3	Mesothelioma	**CHUK P330S, BAP1 fs**	Cytotoxic	4	NA	Routine clinical mechanisms
	4	Head and neck cancer	None identified	Cytotoxic	NA	NA	Routine clinical mechanisms
	5	Cervical Cancer	**PREX2 A1523P, PIK3CA copy number gain**	Cytotoxic	2	NA	Routine clinical mechanisms
**Targeted Therapy (n = 13)**	6	Uterine Cancer	**PIK3CA E542K, STK11 S216F**	STKI – PI3K inhibitor	2	SD	Clinical trial
	7	Non-small cell lung cancer	**PIK3CA E542K**	STKI – PI3K inhibitor	2	SD	Clinical trial
	8	Sarcomatoid renal cell carcinoma	**CCND1 P287T**, YAP1 copy number gain	STKI – CDK inhibitor	4	PD	Clinical Trial
	9	Basal Cell Cancer	**GLI2 RNA log2 = 9.35**	Inorganic compound	2	PD	Off label
	10	Hepatocellular carcinoma	**BAP1 Y94C**	TKI-MKI/HDAC inhibitor	4	PD	Off label
	11	Pancreatic adenocarcinoma	**CSF1R RNA log2 = 4.26, JAK2 RNA log2 = 2.5**	TKI – CSF1R inhibitor	4	PD	Off label
	12	Melanoma	**KIT D816H**	TKI – MKI/KIT inhibitor	3	SD	Off label
	13	Cholangiocarcinoma	**ERRFI1 E384X**	TKI – EGFR inhibitor	3	PR	Off label
	14	Pancreatic adenocarcinoma	**CHUK G288R, GLI3 T183S**	PI	4	PD	Off label
	15	Pancreatic adenocarcinoma	**TGFBR3 N280K, SMURF2 S135N**	PI	4	PD	Off label
	16	Cholangiocarcinoma	Copy number gain in GLI1, FGF3, FGF4, **FRS2, MDM2**, & ERRB2-STARD3 fusion	TKI’s – FGFR inhibitor	4	PD	Off label
	17	Cholangiocarcinoma	FGFR2-MGEA5 fusion	TKI – FGFR inhibitor	4	PR	Off label
	18	Pancreatic adenocarcinoma	**MDM2 copy number gain**	MDM2-I	4	PD	Single patient IND
**No Treatment Received (n = 9)**	19	Cholangiocarcinoma	**PAK1 R371C**	NA	4	NA	NA
	20	Cholangiocarcinoma	FGFR2-BICC1 fusion	NA	4	NA	NA
	21	Pancreatic adenocarcinoma	**NOTCH2 A21T & fs**	NA	4	NA	NA
	22	Testicular cancer	TSSK6 copy number gain, AKT1 copy number loss	NA	4	NA	NA
	23	Extramedullary multiple myeloma	CRBN Q99* & R283K	NA	3	NA	NA
	24	Cholangiocarcinoma	None identified	NA	NA	NA	NA
	25	Pancreatic adenocarcinoma	None identified	NA	NA	NA	NA
	26	Pancreatic adenocarcinoma	None identified	NA	NA	NA	NA
	27	Pancreatic adenocarcinoma	None identified	NA	NA	NA	NA
**Pre-CLIA Pilot Phase (n = 6)**	28	Pancreatic adenocarcinoma	BRCA2 compound heterozygote	NA	3	NA	NA
	29	Gastric adenocarcinoma	FGFR2 amplification	NA	2	NA	NA
	30	Hepatocellular carcinoma	None identified	NA	NA	NA	NA
	31	Cholangiocarcinoma	None identified	NA	NA	NA	NA
	32	Liposarcoma	UHMK1-DDR2 fusion, copy number gain	NA	4	NA	NA
	33	Extramedullary myeloma	CUL4B intronic SNV	NA	4	NA	NA

^*^RECIST response or equivalent data was not available for those patients who did not pursue genomic target directed therapy, NA = not applicable, **BOLD** indicates targets that validated independently in a CLIA certified laboratory, fs = frameshift, SNV = single nucleotide variant, STKI = serine threonine kinase inhibitor, PI3K = phosphatidylinositol 3-kinase, CDK = cyclin dependent kinase, TKI = tyrosine kinase inhibitor, MKI = multi-kinase inhibitor, CSF1R = colony stimulating factor 1 receptor, KIT = KIT proto-oncogene receptor tyrosine kinase, EGFR = epidermal growth factor receptor, FGFR = fibroblast growth factor receptor, HDAC = histone deacetylase, PI = proteasome inhibitor, MDM2-I = MDM2 inhibitor. Level of evidence: 1 = Validated clinical, 2 = Preclinical/limited clinical, 3 = Pre-clinical, 4 = Hypothetical/knowledge based/inferential.

### Molecular Analysis Turnaround

**Figure 2:**
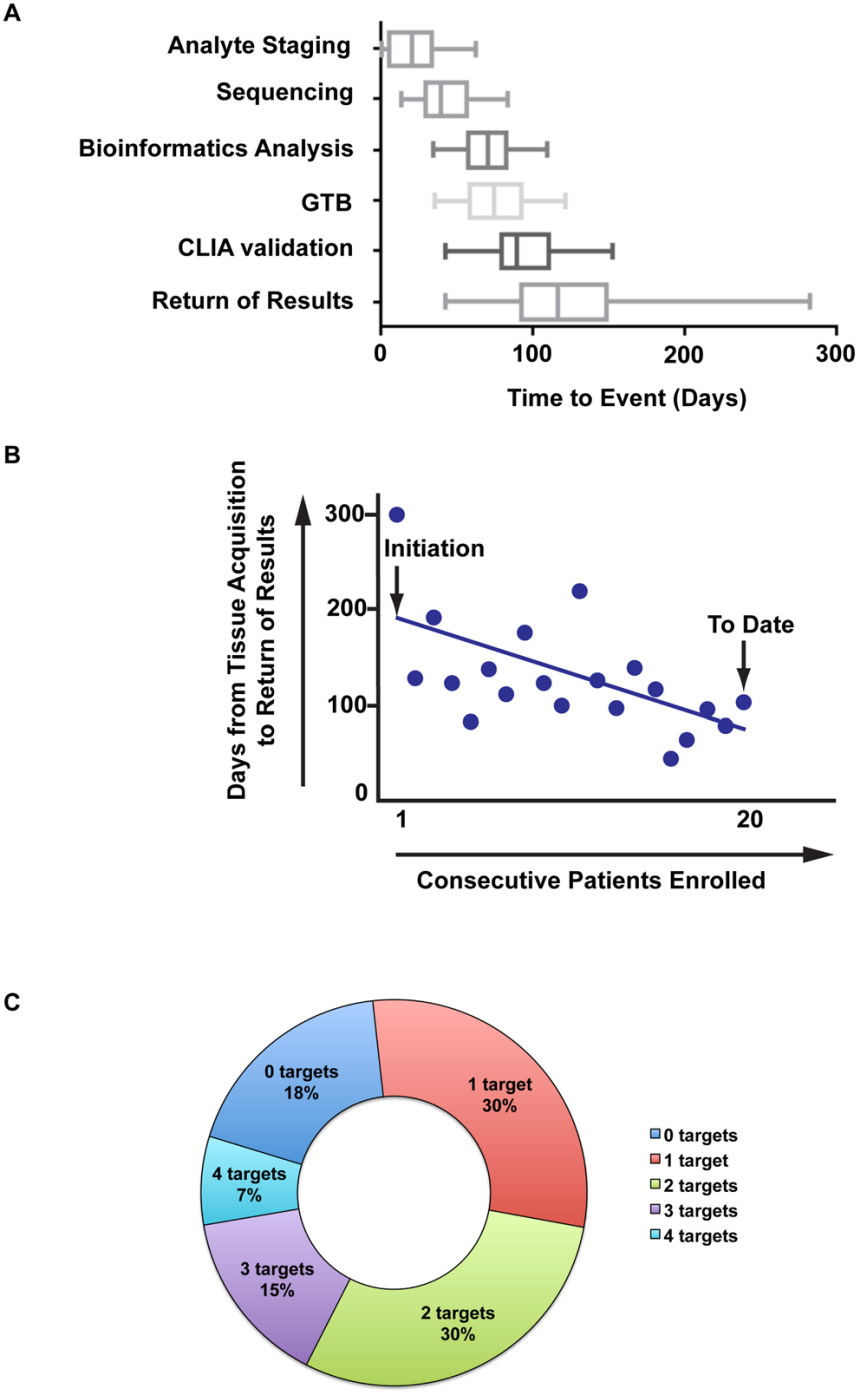
Time required from tissue acquisition to delivery of results to patient and actionable targets identified. (**A**) Range of time it takes to complete each portion of the WGS process. The boxes represent the 25–75th percentile and the line in the centre indicates the median. (**B**) Decrease in time for delivery of results to patient. (**C**) Summary of actionable targets identified per patient.

The median time it took to complete the molecular analyses from tissue acquisition to return of CLIA validated results to the patient was 116 days (Fig. 2A). It should be noted that over the course of the study, there was consistent improvement through a decrement in this parameter (Fig. 2B), as the sequencing and bioinformatics workflows gained efficiencies. With the advancement of sequencing technology, the amount of time required to obtain raw sequencing data has declined significantly (<24 hours).

### Sequencing & Bioinformatics Analyses

Six patients enrolled in the pilot phase of this trial. Half underwent whole genome sequencing (WGS) only, while the other half underwent WGS and whole transcriptome sequencing (RNAseq) (Supplementary Fig. S1). In the CLIA phase, long-insert WGS (li-WGS), whole exome sequencing (WES) and RNAseq were performed on 23 (85%) patients. Analyte quality for DNA and RNA utilised in each phase is presented in Supplementary Table S1. Sequencing statistics for each phase are presented in Supplementary Table S2.

### Identification of Pathogenic Germline Findings

Germline sequencing was primarily used to insure that the genetic alterations were tumour-specific, and not inherited private variants. However, germline sequencing opens the possibility of uncovering unforeseen incidental findings that may be relevant to the patient or their family. We examined incidental findings under guidance by the American College of Medical Genetics (ACMG)^15^. Overall, there were six variants that were classified as pathogenic by two or more submitters within NCBI ClinVar database, including variants in *MSH6* (associated with Lynch Syndrome), *MUTYH* (associated with Colorectal adenomatous polyposis), *NTRK1* (associated with medullary thyroid cancer), *SDHD* (associated with Paraganglioma-pheocromocytoma syndrome), and *TSC1* (associated with Tuberous Sclerosis) (Supplementary Table S3). Two of these variants did not fit criteria for reporting, *NTRK1* was not reported as it is a variant attributed to insensitivity to pain, and *MUTYH* was not reported since a second bi-allelic variant that was known pathogenic was not observed. Variants were determined to be potentially pathogenic if they were predicted deleterious by multiple algorithms including CADD^16^ and Polyphen2^17^, impacted over 75% of transcripts, and were located proximal to the 5′ coding end. Variants predicted to probably predispose to cardiomyopathies and aortic aneurysms were identified in *KCNH2*, *GLA* and *SMAD3*. Additionally, probably or possibly pathogenic variants included an individual with a *BRCA2* variant. Notably, this was a frameshift *BRCA2* variant truncating approximately 50% of the longest canonical transcript within a 29 year-old pancreatic cancer patient with a family history of breast cancer.

### Somatic Aberrations

Somatic variant calling revealed a median of 79 (range 28–8891) potentially functional somatic point mutations per patient. From these variants, 1–3 targetable single nucleotide variants (SNV) per patient were identified in 75% of our cohort (Fig. 2C). The most common mutation types identified were missense (84%) and nonsense (11%) (Supplementary Table S3). A total of 139 aberrations including SNVs, copy number variants (CNVs) and differentially expressed genes were reported to the Clinical GTB (Supplementary Table S4) of which 44 were selected for validation in a CLIA laboratory (Supplementary Table S5). The majority (82%) of these events were validated with an independent method. For events that were more challenging to validate, more than one validation method was employed. SNVs were validated using Sanger sequencing, expression changes were validated by RT-PCR, copy number changes by FISH and protein expression confirmed by IHC.

### Efficacy of Integrated Genomic Profiling

In order to evaluate the efficacy of this comprehensive sequencing approach, we took four commercially available cancer panels (FoundationOne®, Caris MI Profile®, PGDx® and Paradigm®), that included 252 genes at the time IGP was conducted, and compared the 139 aberrations reported to the Clinical Genomics Tumour Board against each of these panels to determine if they could have been detected by a panel test alone. Fifty percent of the genes with reported somatic events (SNVs, CNVs and/or differential expression) were not present on any of the panels. When the mutations present in genes found on commercial panel tests were evaluated more closely, it was observed that while 7% of these DNA mutations should be identified by a panel, differential expression of the gene would have not been detected due to the panel only testing DNA. An additional 8% of the reported events had only differential expression of the gene, without evidence of mutation in the DNA at all, thus would have been missed entirely by the DNA only panels targeting those genes. Of the 13 patients who received targeted therapy, six of them had tumours with somatic mutations that would not have been detected by these panel tests.

**Figure 3:**
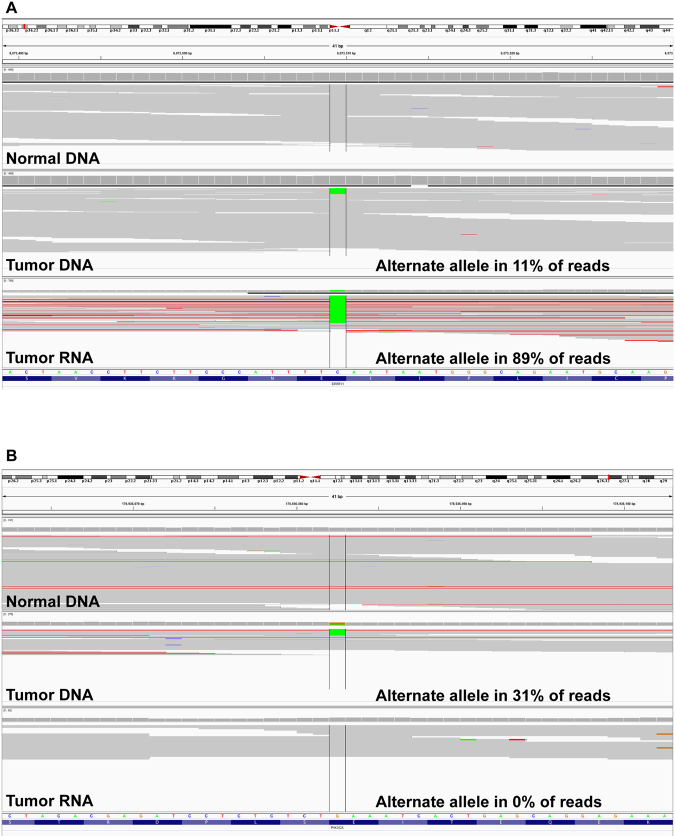
(**A**) Presence and (**B**) absence of alternate alleles in RNA.

This integration of WES, li-WGS and RNAseq into the analysis allowed for the assembly of a more complete picture of the mutational landscape of each patient. For example, in one tumour (Case 13, Table 2) the alternate allele in the DNA represented only 11% of the reads while in the RNA, the alternate allele was present in 89% of the RNA reads suggesting enrichment of the mutant transcript (Fig. 3A). Evaluation of DNA alone would not have recognised the significant contribution of this tumour cell specific expression. In contrast, in a second instance (Case 6, Table 2), the presence of the mutation in the tumour DNA is clear with 31% of the reads containing the alternate allele, but the alternate allele is completely absent in the RNA reads (Fig. 3B). Interestingly, 45% of the SNV and CNV observed in DNA also demonstrated the presence of the alternate allele in the RNA and/or demonstrated differential expression in the RNA. Only one case did not have RNAseq conducted and thus there was no RNA evidence to query. Other factors can also contribute to differing DNA and RNA allelic fractions such as tumour heterogeneity and technical issues introduced during the sequencing process. To minimize these factors DNA and RNA were extracted from the same tissues, quality checks were implemented during the sequencing process and all variants were manually inspected prior to inclusion in the final variant report.

### Optimisation of Target Prioritisation Through Integrated Genomic and Transcriptomic Analysis

Integration of whole genome, whole exome and whole transcriptome data allowed for enhanced target prioritisation. Case 13 and Case 17 highlight this aspect of the study. In the evaluation of Case 13 (Table 2) using WES, li-WGS and RNAseq, it was noted that while only 11% of WES reads had mutant *ERRFI1*, 89% of the RNAseq reads had the mutant transcript (Fig. 3A). The mutant transcript enrichment of *ERRFI1* allowed for the GTB to prioritise and nominate ERRFI1 as a therapeutic target with intervention potential with EGFR inhibitors. The patient was treated with erlotinib and achieved a partial response by RECIST criteria.

**Figure 4:**
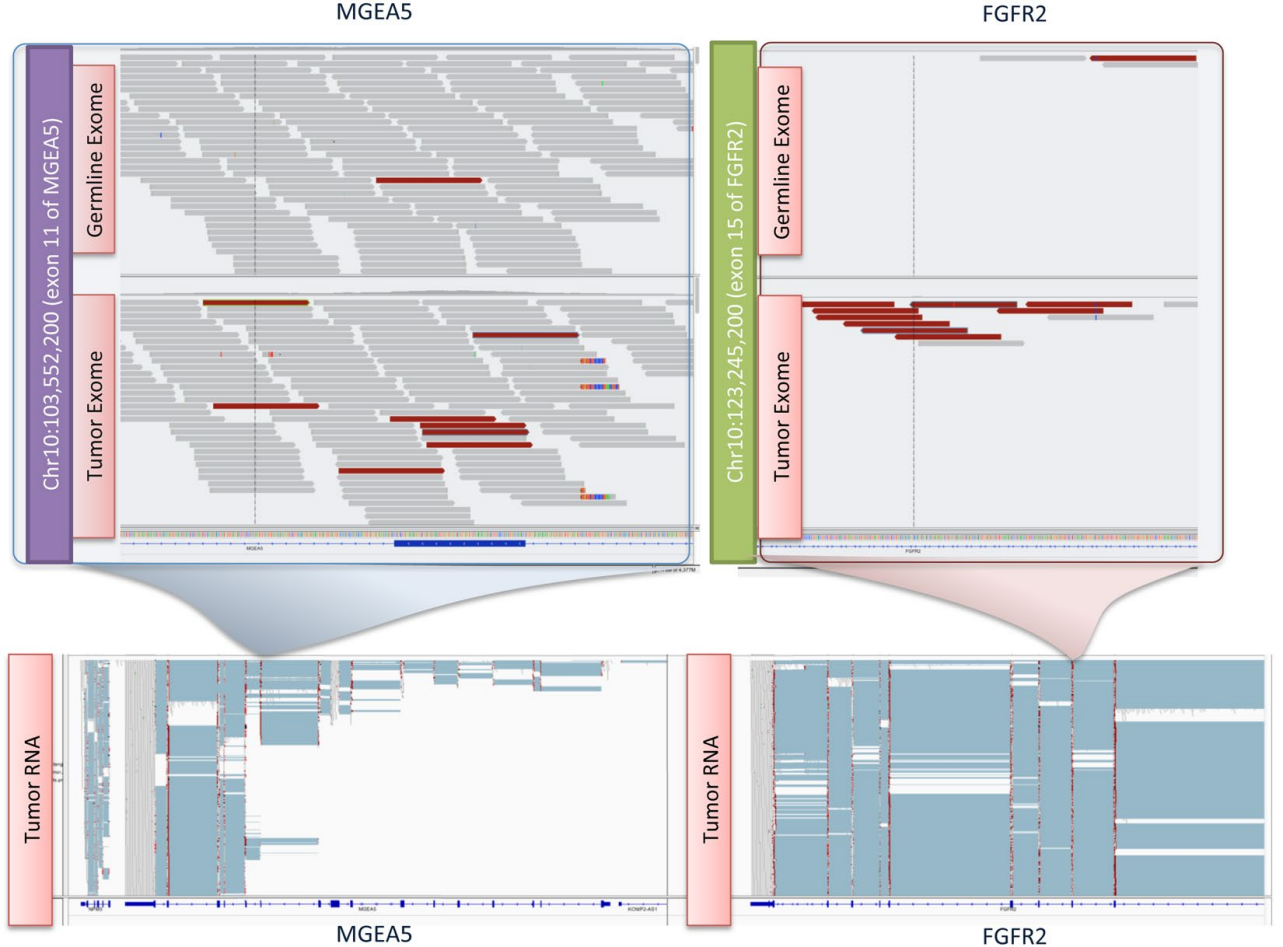
Visualisation of FGFR2-MGEA5 fusion in the Integrative Genomics Viewer (IGV).

Assessment of Case 17 highlighted the value of using multiple platforms for identification of potential therapeutic targets. A fusion in FGFR2-MGEA5 was discerned both in the RNAseq and the WES analyses (Fig. 4). The concordant nature of these findings allowed for its prioritisation as a drug target.

## Discussion

Despite the explosion in utilisation of NGS towards discovery efforts in virtually all biological and medical disciplines, currently there are no whole genome/transcriptome assays that have achieved analytical validation levels and reproducibility that would support their implementation as stand-alone assays that do not require secondary confirmation with approaches such as capillary sequencing, PCR, FISH or IHC. Guidelines for standardisation and quality metrics have been proposed by the Next-generation sequencing: Standardisation of Clinic Testing group (Nex-StoCT)^18^ and ACMG^19^. The dynamic nature of this space is exemplified by the recent guidance by the FDA pertaining to laboratory developed tests such as NGS assays^20^. These considerations highlight the importance of conducting integrated whole genome/transcriptome sequencing for elucidation of therapeutic targets in a CLIA enabled workflow.

Despite their inability to allow for whole genome analysis, panel-based approaches currently retain technical and logistical advantages that are desirable including ability to achieve greater depth of coverage for individual variants allowing for identification of low frequency events, shorter TRR (typically less than 2 weeks) and CLIA-CAP compliant analytical test attributes not requiring secondary validation.

A recent study^9^ has highlighted the high false positive rate of variant calls associated with panel-based approaches, particularly those that do not utilise germline comparators. This study illustrates the advantages that application of Integrated Genomic Profiling (IGP) may confer over panel-based approaches in a real time clinical setting. These include optimisation of target prioritisation and comprehensive variant assessment that inherently uncovers novel putative targets. Furthermore, inclusion of germline variant analysis enables identification of germline variants amenable to therapeutic targeting and communication with patients regarding hereditary risk. A weakness of IGP is the potential for false negative variant calls due to a lower coverage depth than that provided by panel tests. While this and prior studies^3,5,21^ have delineated a platform for implementation of integrated whole genome analysis in the clinic, significant challenges remain that preclude large-scale implementation.

TRR, from time of biopsy/tissue access, in our study was a median of 116 days. While this still allowed for 20 (69%) patients in the CLIA phase to achieve return of results and 13 (65%) to receive eventual molecular profiling based therapy, TRR durations of greater than two weeks are unlikely to eventually have broad, meaningful clinical impact. Process improvements over the course of our study resulted in a consistent decrement of TRR (Fig. 2B). Availability of faster sequencing platforms over the course of the study reduced sequencing time from ~3 weeks to ~24 hours. Several additional areas were identified for process improvement to further reduce TRR. First, improvement in bioinformatics tools/workflows and computational platforms are needed to rapidly process sequencing data^22^. Second, it is vital to the future ability of massively parallel whole genome, exome and transcriptome sequencing that it be conducted in a CLIA compliant environment that does not require secondary confirmation with approaches such as capillary sequencing or PCR, as was undertaken in our study. Finally, end-user operated biointelligence tools that operate freely from the constraints of scheduling and personnel availability that are inherent with human multi-disciplinary GTBs, are necessary to further streamline TRR.

Integration of NGS data from multiple platforms has been undertaken extensively in efforts such as TCGA (http://cancergenome.nih.gov/) and ICGC (https://icgc.org)^1,23^. Clinical application of such integrated approaches has been limited thus far^3,5^. Prioritisation and nomination of aberrations as putative drug targets can be challenging in the absence of recurrently identified aberrations, supporting functional or clinical efficacy data. IGP can facilitate target prioritisation and provide detection of variants in genes not evaluated in panel testing. While the use of allelic fraction in RNA versus DNA as a predictor of response is investigational, the utility of integration of data from multiple NGS platforms (WES, li-WGS and RNAseq) is illustrated through the demonstration of mutant transcript enrichment (Case 13, *ERRFI1* mutation, Fig. 3A) as well as multi-platform concordance or discordance of alterations (Case 17, FGFR2-MGEA5 fusion, Fig. 4). Furthermore, data integration can also aid in the identification of putatively hyperselected aberrations such as a gene that is both amplified and somatically mutated or homozygously deleted.

Multiple studies have now shown that tumours in patients with advanced cancers are genomically heterogeneous at intratumoural, intrapatient-spatial and temporal-longitudinal levels^24,25^. While it has been essential in prior pilot studies and our study to establish the safety and feasibility of application of integrated genomic analysis, these efforts have utilised biopsy material from limited, discrete anatomical regions in individual patients. Given that decisions made from genomic profiling of spatially discrete regions in an individual carry an inherent inability to capture the comprehensive clonal profile of their cancer, future efforts would greatly benefit from multi-region sequencing. Deployment of computational^26^ and flow-sorting based approaches^27,28^ would help enable tumour heterogeneity assessment. Finally, recent novel applications of NGS in the context of circulating DNA in plasma (ctDNA), has opened up the possibility of clinical application of “liquid biopsies” which would not be burdened with the clinical safety risks when evaluated serially compared to multiple tissue/organ biopsies^29,30^.

Drug access, which constitutes the terminal step in a paradigm for profiling for therapeutic intent, remains a major challenge. A recently initiated whole genome profiling study in melanoma in BRAF wild type patients^31^ and the NCI Molecular Analysis for Therapy Choice (MATCH) solid tumour study both illustrate the importance of having a multi-arm design which provide patients with potential access to a broad array of targeted therapies. From a clinical trial design perspective, single arm studies will be primarily hypothesis generating in nature and more robust designs such as the Southwest Oncology Group Lung-MAP squamous cell lung cancer trial^32^ that employ control arms will more confidently ascertain the benefit of genome profiling based approaches.

Of note, in our study, application of “actionable” findings resulted in no discernible clinical benefit in 8 (62%) patients. This low response rate is akin to that of other precision medicine studies^7,10,33–36^ and is multi-factorial due to challenges in tissue acquisition, time to return of results, drug access and clinical trial eligibility. While there would be consideration of factors such as advanced/late stage of disease where application of any intervention would likely be unsuccessful and paucity of potent therapeutics for therapeutically challenging drug targets (e.g. KRAS), these preliminary observations may illustrate a critical need to both capture and share such negative information. It is imperative that Clinical Utility Indices (CUIs) be developed that can rapidly convey composite information encompassing druggability/likelihood of clinical benefit that is context dependent (e.g. disparity of connotations of BRAF mutations in melanoma versus colorectal cancer) and associated level of evidence ranging from pre-clinical only to definitive clinical studies.

In conclusion, the feasibility of IGP in a CLIA enabled workflow has been successfully demonstrated in a consistent fashion in patients with complex disease diagnosis through germline evaluation. Although limitations to broad applicability such as TRR and drug access remain, this study represents one of the largest efforts to date of integrated, CLIA-enabled IGP for therapeutic target assessment in patients with advanced cancer and demonstrates the value of IGP in advanced cancer care. It also provides a contextual framework to incorporate other genomic analyses such as mi-RNA sequencing, epigenomic assessment and long noncoding RNA (lncRNA) evaluation into the workflow as therapeutics utilising these approaches enter the clinic and their role in therapeutic response prediction is better defined. Results from larger ongoing efforts such as the Can-Seq initiative^37^ are also eagerly awaited.

## Methods

### Ethics Statement And Sample Collection

Clinical information was assimilated from patient records at the Mayo Clinic. Informed consent was obtained for each patient on two ongoing research protocols approved by the Mayo Clinic Institutional Review Board (10-006180 [advanced cancers] and 10-002879 [rare cancers]). Patients were confirmed to have met the following eligibility criteria: life expectancy of at least three months, diagnosis of histologically or cytologically confirmed advanced incurable cancer, at least 18 years of age, a good candidate for biopsy or surgical procedure to obtain tissue, and no uncontrolled concurrent illness. Clinicopathological features collected included: age, gender, stage, histological grade, sites of metastasis, tumour sample assessment for overall cellularity/necrosis as well as percent tumour cellularity and prior therapies. Methods were carried out in accordance with the relevant institutional guidelines.

Tumour tissue was acquired by routine clinical methods encompassing imaging-guided needle core biopsies and surgical procedures (Table 1). Tissue specimens were collected fresh frozen and maintained below −80 °C until nucleic acid extraction. A board certified pathologist experienced in biospecimen studies evaluated a portion of each specimen to confirm the presence of tumour, degree of necrosis and percent cellularity. Germline peripheral blood mononuclear or buccal swab samples were collected and maintained at ambient temperature until nucleic acid extraction.

### Nucleic Acid Isolation

DNA and RNA were extracted from tumour samples in a Clinical Laboratory Improvement Amendments (CLIA) compliant environment while the normal samples were extracted in a research laboratory. In order to obtain sufficient material for sequencing and subsequent CLIA validation, nucleic acids extracted from multiple tissue cores of a single patient were pooled. DNA and RNA with a 260/280 of 1.8–2.1 and a 260/230 of 2.0–2.2 were considered of sufficient quality to proceed with library preparation. For DNA only, an aliquot was run on a gel to determine if the sample was degraded. For RNA only, a RIN >7 was preferred for RNA sequencing.

### Sequencing

Prior to sequencing, library quality was checked by assessing the yield (minimum of 2 nM) and compared with prior yields to identify potential problems with the sample or reagents. A Bioanalyzer® trace was also evaluated for overamplification or unexpected phenotypes. Long-insert whole genome, whole exome and RNA sequencing libraries were prepared using previously published methods^38,39^. All samples were sequenced on the Illumina HiSeq 2000 or 2500.

### Alignment, Somatic Variant Calling and Fusion Detection

Alignment, variant calling and fusion detection were conducted as previously described^39^. Final somatic point mutations for functional analysis exceeded a quality score of 20 as calculated by Seurat 2.5, showed at least 1 read support of the mutation in both read directions, were covered by at least 20 reads overall in both tumour and normal, and were predicted functional by snpEff 3.3 for transcripts documented as coding for protein annotated using Ensembl 71 reference definitions. Genes exhibiting a breakpoint indicating pretense of a structural variant contained at least 10 anomalous read-pairs supporting the break requiring paired reads map within 2 kb windows each and requiring that a window immediately prior or before showed a coverage level within the normal to be less than 2 standard deviations from the genome. Focal amplifications and deletions were defined as copy number changes greater than a magnitude of 0.75, and less than 15 megabases in length, noting that longer events were generally considered chromosomal level gains or losses.

### Germline Variant Calling

Germline base call files were generated by the Illumina HiSeq2000 RTA module during sequencing and were converted to demultiplexed fastq files using CASAVA 1.8.2 (Illumina, San Diego, CA). Quality filtered reads from exome sequencing were aligned to NCBI reference GRCh37.62 with BWA 0.6.2-r126^40^. Binary alignment files were converted and coordinate sorted into the standard BAM format using samtools 0.1.18^41^. Aligned reads were realigned around short insertion and deletions and duplicate reads were filtered using Picard 1.79 (http://picard.sourceforge.net/). This followed aligned base quality recalibration with GATK 2.2^42^. Flowcell lane level sample BAMs were then merged with Picard 1.79 if samples were sequenced across multiple lanes. Variant calling was done by UnifiedGenotyper and genotype quality recalibrated using VariantRecalibrator described in the best practice methods of GATK 2.2^43^. Pathological variants were determined for genes within ACMG guidelines, whereby pathogenicity was either defined loss of function variant such as by a stop-gain or frameshift, or previously documented using ClinVar designation of pathogenic from the ClinVar VCF file-date 20130507^15^.

### Validation

Identified targets were validated in a CLIA setting utilising reverse transcription-polymerase chain reaction (RT-PCR), capillary sequencing, fluorescent *in-situ* hybridisation (FISH) and/or immunohistochemistry (IHC).

### Treatment Assignment Algorithm

The process summary is illustrated in Supplementary Fig. S2. Disparate gene state annotation data were assembled into a relational gene centric characterisation database. This allowed for compilation of all available genomic information from multiple platforms (exome, whole genome and transcriptome) to facilitate efficient and effective access of relevant data for subsequent knowledge mining. A hypergraph-based scalable computational framework was used to structure a biointelligence platform (BIP) that allowed for representation of multi-lateral, multi-scalar and multi-dimensional representation of these complex data sets^43,44^. The BIP allowed for integration of data from many publicly available databases querying information about: sequencing and gene annotation, molecular pathways, drugs and therapeutics, clinical trials, gene-molecule context and drug repurposing (Supplementary Table S6). In addition, commercially available tools Ingenuity Pathway Analysis® and GeneGo Metacore® were employed. Additional data filtering utilised publicly available literature review, expert input and incorporation of heuristic/iterative knowledge.

The initial prioritised list of “putative” targets for each patient was discussed at a multi-disciplinary genomics tumour board (GTB) comprising experts from genomics, bioinformatics, medical oncology, haematology, ethics, medical genetics, pharmacogenomics, molecular biology and pharmacology. GTB meetings were convened upon availability of data for individual patients. Individuals with expertise outside of the core GTB membership were included on an *ad hoc* basis when insight/knowledge feedback regarding specific signalling pathways or diseases was required for decision-making. Prioritisation of “actionable” targets also entailed utilisation of level of evidence tiers (validated clinical > pre-clinical only > hypothetical/knowledge-based/inferential). If applicable, combination therapy was recommended when alterations from multiple pathways in a specific case would confer potential for escape if a single agent was used, and if safety data from the suggested combination of drugs was available from previously conducted Phase IB/II/III studies.

Upon completion of discussion of individual cases at the GTB, a finalised list of “actionable” targets was constructed for CLIA validation prior to generation of a formal report that was delivered to the treating physician and integrated into the electronic medical record. Upon availability of the “actionable” target list/report the treating physician assigned targeted therapy. The treating physician reviewed the prioritised “actionable” targets for individual patients to ascertain any contraindications arising from clinical characteristics/co-morbidities and assessment of degree of potential benefit compared to benefit with clinically available agents, to generate a list of “intervenable” targets.

The widest available spectrum of drug access mechanisms was applied to the “intervenable” targets. These included: on-label and off-label use of FDA approved drugs, clinical trials and single patient INDs. Patients who eventually received genomic profiling directed therapy were followed for assessment of preliminary evidence of anti-tumour efficacy using standard approaches such as response criteria in solid tumours (RECIST v1.1)^45^. Toxicity was monitored using NCI Common Terminology Criteria v4.03. Patients for whom genomic profiling directed therapy was inaccessible despite exhaustive efforts to secure drug access were treated with clinically available therapies or supportive care per the direction of the patient’s treating oncologist.

## Electronic supplementary material

Supplementary Materials
