# Supplementary material for: Clinical Implementation of Integrated Genomic Profiling in Patients with Advanced Cancers
Source: Sci Rep. 2016 Dec 23;6:25. doi: 10.1038/s41598-016-0021-4 (PMC5431338; doi:10.1038/s41598-016-0021-4)
Supplement: Supplementary file 1 — Supplementary Materials [file 41598_2016_21_MOESM1_ESM.pdf]

Clinical Implementation of Integrated Genomic Profiling in Patients with Advanced Cancers

Authors

Mitesh J. Borad[1,2,3,*], Jan B. Egan[3], Rachel M. Condjella[2], Winnie S. Liang[4], Rafael Fonseca[1,2,3], Nicole R. Ritacca[2], Ann E. McCullough[5], Michael T. Barrett[2,4], Katherine S. Hunt[1], Mia D. Champion[3,6], Maitray D. Patel[7], Scott W. Young[7], Alvin C. Silva[7], Thai H. Ho[1,2,3], Thorvardur R. Halfdanarson[1,2,3], Robert R. McWilliams[3,8], Konstantinos N. Lazaridis[3], Ramesh K. Ramanathan[1,2], Angela Baker[4], Jessica Aldrich[4], Ahmet Kurdoglu[4], Tyler Izatt[4], Alexis Christoforides[4], Irene Cherni[4], Sara Nasser[4], Rebecca Reiman[4], Lori Cuyugan[4], Jacquelyn McDonald[4], Jonathan Adkins[4], Stephen D. Mastrian[4], Riccardo Valdez[5], Dawn E. Jaroszewski[9], Daniel D. Von Hoff[4], David W. Craig[4], A. Keith Stewart[1,2,3], John D. Carpten[4], Alan H. Bryce[1,2,3]

Affiliations:

* Corresponding author

1. Division of Hematology/Oncology Mayo Clinic, Scottsdale, AZ, U.S.A.
2. Mayo Clinic Cancer Center, Scottsdale, AZ, U.S.A.
3. Center for Individualized Medicine, Mayo Clinic, Rochester, MN, U.S.A.
4. Translational Genomics Research Institute, Phoenix, AZ, U.S.A.
5. Department of Pathology, Mayo Clinic, Scottsdale, AZ, U.S.A.
6. Department of Biomedical Statistics and Informatics, Mayo Clinic, Scottsdale, AZ, U.S.A

7. Department of Radiology, Mayo Clinic, Scottsdale, AZ, U.S.A.
8. Mayo Clinic Cancer Center, Rochester, MN, U.S.A.
9. Department of Cardiovascular Surgery, Mayo Clinic, Scottsdale, AZ, U.S.A.

Corresponding Author:

Mitesh J. Borad

Mayo Clinic in Arizona

13400 E Shea Blvd

Scottsdale, AZ 85259

Office: 480-342-4800

Fax: 480-301-4675

borad.mitesh@mayo.edu

Supplementary Figure S1. Sequencing methods utilised

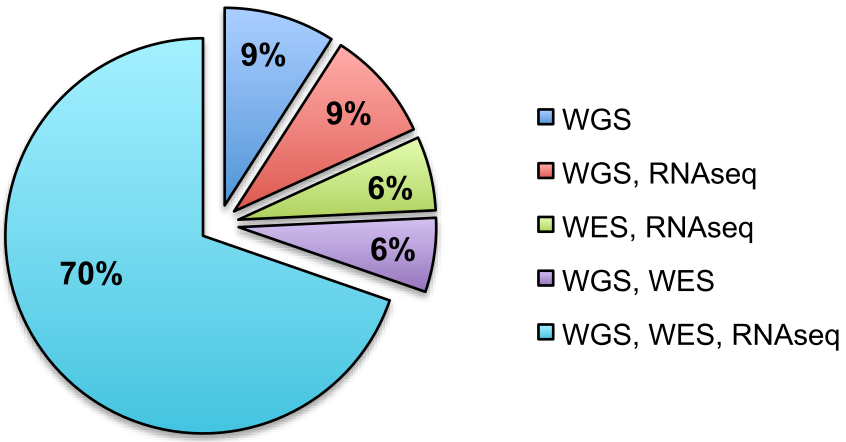

	Sequencing combination used	Number
Pilot phase	WGS	3
	WGS, RNAseq	3
CLIA phase*	WES, RNAseq	2
	WGS, WES	2
	WGS, WES, RNAseq	23

* WGS was long-insert whole genome

Supplementary Fig. S2. Workflow from initial consultation to treatment

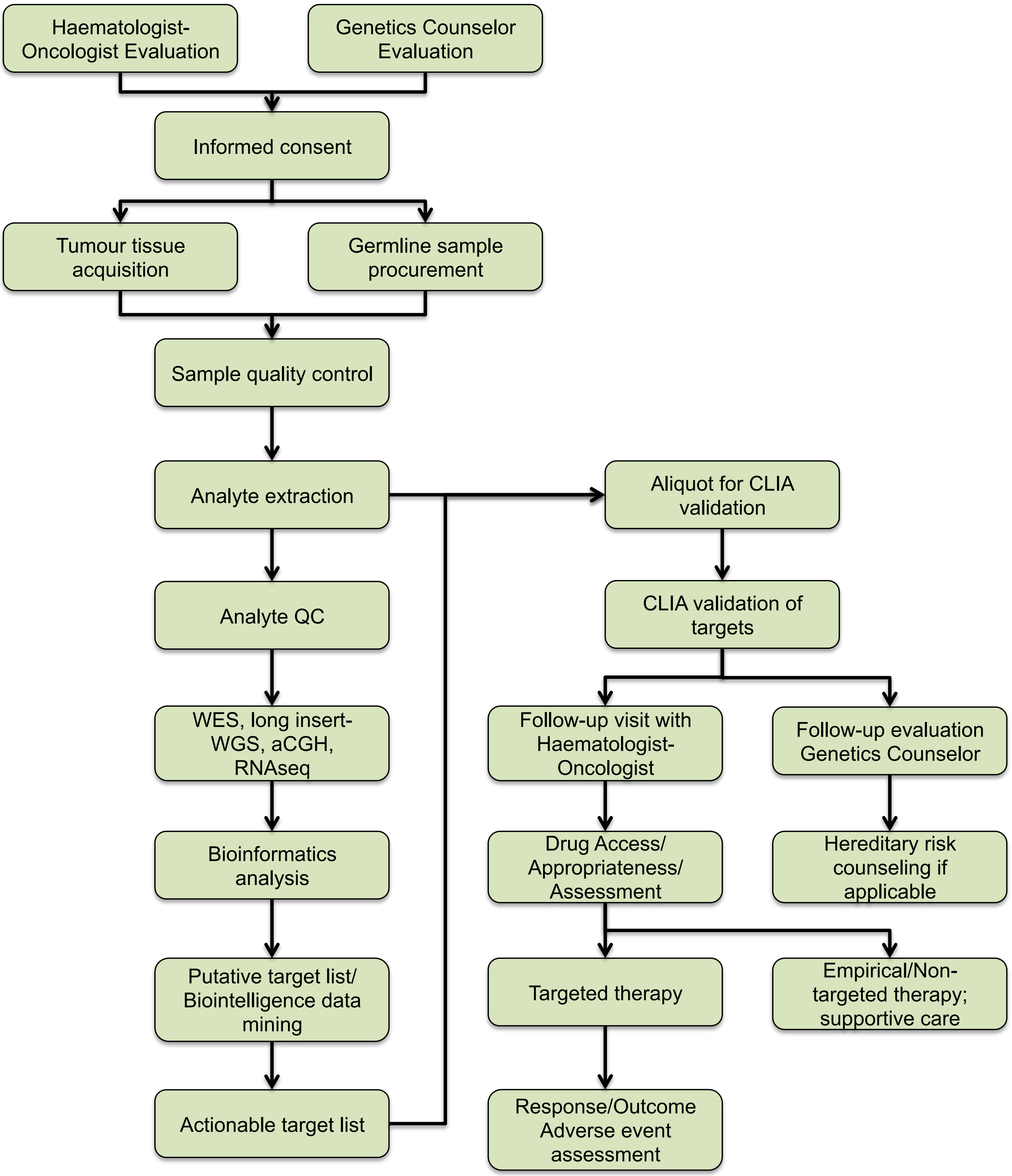

Supplementary Table S1. Summary of DNA and RNA quality

			Median RIN score (range)	Median 260/280 ratio (range)
Pilot phase	RNA	Normal	N/A	N/A
		Tumor	8.1 (4.0-9.3)	1.99 (1.94-2.04)
	DNA	Normal	N/A	1.86 (1.78-1.91)
		Tumor	N/A	1.87 (1.68-1.9)
CLIA phase	RNA	Normal	7.3 (0-10)	2.09 (1.95-2.11)
		Tumor	6.8 (1.8-9.5)	2.04 (0.65-4.9)
	DNA	Normal	N/A	1.87 (1.70-2.08)
		Tumor	N/A	1.88 (-0.26-4.93)

Supplementary Table S2. Sequencing statistics

		All				Clinical Genomics Tumor Board				Pilot Study			
	**Tumor**	**Count**	**Average**	**Min**	**Max**	**Count**	**Average**	**Min**	**Max**	**Count**	**Average**	**Min**	**Max**
Whole Genome	Physical coverage	31	44	4	149	25	34	4	73	6	89	31	149
	Aligned Reads (10^6)	31	618.7	26.9	3121.2	25	306.9	26.9	1166.4	6	1917.5	1204.4	3121.2
	Aligned Bases (10^9)	31	59.9	2.2	312.1	25	28.5	2.2	95.1	6	191.0	111.4	312.1
	Insert size	31	666	159	1003	25	757	253	1003	6	286	159	375
	Genome size (10^9)	31	3.09	3.09	3.09	25	3.09	3.09	3.09	6	3.09	3.09	3.09
	Base Coverage	31	19	1	101	25	9	1	31	6	62	36	101
	**Normal**												
	Physical coverage	31	45	13	130	25	36	13	71	6	84	26	130
	Aligned Reads (10^6)	31	595.3	101.0	2788.3	25	298.8	101.0	1014.9	6	1830.9	1052.3	2788.3
	Aligned Bases (10^9)	31	57.7	8.3	278.8	25	27.9	8.3	83.1	6	182.2	96.8	278.8
	Insert size	31	691	155	931	25	789.92	224	931	6	280	155	344
	Genome size	31	3.09	3.09	3.09	25	3.09	3.09	3.09	6	3.09	3.09	3.09
	Base Coverage	31	19	3	90	25	9	3	27	6	59	31	90
Exome	**Tumor**												
	Aligned Reads (10^6)	27	160.0	70.8	219.1	27	160.0	70.8	219.1	N/A	N/A	N/A	N/A
	Percent reads aligned	27	98%	93%	99%	27	98%	93%	99%	N/A	N/A	N/A	N/A
	Aligned Bases (10^9)	27	14.7	7.3	23.1	27	14.7	7.3	23.1	N/A	N/A	N/A	N/A
	Mean Coverage of Targets	27	106	32	210	27	106	32	210	N/A	N/A	N/A	N/A
	Target Bases at 10X	27	93%	84%	96%	27	93%	84%	96%	N/A	N/A	N/A	N/A
	Target Bases at 20X	27	88%	76%	93%	27	88%	76%	93%	N/A	N/A	N/A	N/A
	Target Bases at 30X	27	82%	54%	90%	27	82%	54%	90%	N/A	N/A	N/A	N/A
	**Normal**												
	Aligned Reads (10^6)	27	183.7	110.7	259.7	27	183.7	110.7	259.7	N/A	N/A	N/A	N/A
	Percent reads aligned	27	98%	94%	99%	27	98%	94%	99%	N/A	N/A	N/A	N/A
	Aligned Bases (10^9)	27	16.5	10.4	25.5	27	16.5	10.4	25.5	N/A	N/A	N/A	N/A
	Mean Coverage of Targets	27	114	32	249	27	114	32	249	N/A	N/A	N/A	N/A
	Target Bases at 10X	27	94%	91%	96%	27	94%	91%	96%	N/A	N/A	N/A	N/A
	Target Bases at 20X	27	90%	77%	94%	27	90%	77%	94%	N/A	N/A	N/A	N/A
	Target Bases at 30X	27	84%	55%	92%	27	84%	55%	92%	N/A	N/A	N/A	N/A

RNA

**Tumor**												
Aligned Reads (10^6)	28	183.3	47.0	401.5	25	176.5	47.0	401.5	3	239.7	150.6	287.1
Aligned Bases (10^9)	28	15.4	3.7	32.9	25	15.2	3.7	32.9	3	16.7	7.5	28.5
% Ribosomal Bases	25	15.9%	0.4%	55.9%	25	15.9%	0.4%	55.9%	0	N/A	N/A	N/A
% Coding Bases	28	38.4%	7.4%	59.0%	25	38.0%	7.4%	59.0%	3	41.8%	35.3%	46.5%
% UTR Bases	28	30.6%	16.0%	41.6%	25	29.8%	16.0%	39.1%	3	36.9%	31.4%	41.6%
% Intronic Bases	28	11.1%	3.3%	26.0%	25	11.2%	3.3%	26.0%	3	10.6%	7.3%	13.1%
% Intergenic Bases	28	5.6%	2.2%	11.5%	25	5.0%	2.2%	11.2%	3	10.7%	10.0%	11.5%
% MRNA Bases	28	69.1%	23.5%	93.1%	25	68.0%	23.5%	93.1%	3	78.7%	76.9%	81.2%
**Normal**												
Aligned Reads (10^6)	13	139.8	58.9	208.7	13	139.8	58.9	208.7	N/A	N/A	N/A	N/A
Aligned Bases (10^9)	13	11.6	4.5	16.6	13	11.6	4.5	16.6	N/A	N/A	N/A	N/A
% Ribosomal Bases	13	20.0%	0.5%	34.8%	13	20.0%	0.5%	34.8%	N/A	N/A	N/A	N/A
% Coding Bases	13	35.2%	14.1%	83.4%	13	35.2%	14.1%	83.4%	N/A	N/A	N/A	N/A
% UTR Bases	13	20.5%	14.5%	38.8%	13	20.5%	14.5%	38.8%	N/A	N/A	N/A	N/A
% Intronic Bases	13	19.9%	0.6%	43.2%	13	19.9%	0.6%	43.2%	N/A	N/A	N/A	N/A
% Intergenic Bases	13	4.3%	1.0%	7.7%	13	4.3%	1.0%	7.7%	N/A	N/A	N/A	N/A
% MRNA Bases	13	55.8%	33.7%	97.8%	13	55.8%	33.7%	97.8%	N/A	N/A	N/A	N/A

N/A = not applicable

Supplementary Table S3. Germline variants and somatic mutations.

A.

	SOMATIC			
	All		Reported	
	All patients	Median per patient	All patients	Median per patient
**Nonsynonymous coding**	11823	64	65	2
**Frameshift**	305	4	3	1
**STOP gained**	1525	5	5	1
**Codon insertion**	11	1	1	1
**Codon deletion**	21	1.5	1	1
**Codon change plus codon deletion**	8	1	0	0
**Codon change plus codon insertion**	8	1	0	0
**Splice site donor**	89	2	0	0
**START lost**	34	1	0	0
**Splice site acceptor**	166	3	2	2
**STOP lost**	23	1	0	0
**Deletion**	0	0	0	0
**START gained**	0	0	0	0
**Insertion**	0	0	0	0

B. Summary of functional categories of all germline mutations in ACMG listed genes among genes surviving filtering including removing genes outside that are paralogous or otherwise homologous to other regions and with depth below 10, or adjusted quality score below 3-0. (ACMG genes were *BRCA1, BRCA2, TP53, STK11, MLH1, MSH2, MSH6, PMS2, APC, MUTYH, VHL, MEN1, RET, NTRK1, RB1, SDHD, SDHAF2, SDHC, SDHB, TSC1, TSC2, WT1, NF2, COL3A1, FB1, TGFBR1, TGFBR2, SMAD3, ACTA2, MYH11, MYBPC3, MYH7, TNNT2, TNNI3, TPM1, MYL3, ACTC1, PRKAG2, GLA, MYL2, LMNA, RYR2, PKP2, DSP, DSC2, TMEM43, DSG, KCNQ1, KCNH2, SCN5A, LDLR, APOB, PCSK9, RYR1, CACNA1C*). Probable pathogenic were those variants that were deleterious by intersection of CADD scores greater than 20, deleterious by FATHMM, Deleterious for majority of transcripts by polyphen2, Deleterious for majority of transcripts by MutationTaster

	**Pathogenic**	**Probable**	**Grand Total**
BRCA2		2	2
c.3975_3978dupTGCT(p.Ala1327fs)		1	1
c.7878G>C(p.Trp2626Cys)		1	1
GLA		1	1
c.937G>T(p.Asp313Tyr)		1	1
KCNH2		3	3
c.1598T>G(p.Val533Gly)		3	3
MSH6	2	1	3
c.1508C>G(p.Ser503Cys)		1	1
c.2633T>C(p.Val878Ala)	1		1
c.3261dupC(p.Phe1088fs)	1		1
MUTYH	1		1
c.1187G>A(p.Gly396Asp)	1		1
NTRK1	1		1
c.2339G>A(p.Arg780Gln)	1		1
RYR1		7	7
c.11654G>A(p.Arg3885Gln)		1	1
c.13513G>C(p.Asp4505His)		1	1
c.1661A>G(p.Glu554Gly)		2	2
c.5000G>A(p.Arg1667His)		1	1
c.7025A>G(p.Asn2342Ser)		1	1
c.9262G>A(p.Val3088Met)		1	1
SDHD	1		1
c.34G>A(p.Gly12Ser)	1		1
SMAD3		3	3
c.1222G>T(p.Asp408Tyr)		3	3
TSC1	1		1
c.2194C>T(p.His732Tyr)	1		1
TSC2		1	1
c.1747G>A(p.Ala583Thr)		1	1
**Grand Total**	**6**	**18**	**24**

Supplementary Table S4. Reported mutation types

Mutation type	Total
SNV	67
CNV	27
Differential expression	23
CNV + differential expression	5
Fusion	4
Frameshift	3
Structural variant	3
Fusion + CNV	2
Inframe Insertion	2
Altered splice site	1
Differential expression + SNV	1
Deletion	1

SNV = single nucleotide variant, CNV = copy number variation

Supplementary Table S5. CLIA validation summary

	Mutation type	Total	Range
**Number of targets to validate**	**Total**	**44**	**0-4**
	SNV	25	
	CNV	10	
	Differential expression	7	
	Frameshift	2	
**Number of targets validated**	**Total**	**36**	**0-4**
	SNV	24	
	CNV	5	
	Differential expression	6	
	Frameshift	1	
**Target source**	DNA only	34	0-5
	RNA only	8	0-2
	DNA & RNA	1	0-1
**Validation method***	Sanger sequencing	27	0-3
	Quantitative PCR	16	0-4
	IHC	4	0-2
	FISH	1	0-1

*Totals are not equal as some mutations were validated by multiple methods (e.g. Sanger sequencing to confirm the point mutation and qPCR to assess expression).

Supplementary Table S6. Publically available databases utilized in biointelligence platform

Database	URL	Reference
**Sequencing and gene annotation**		
International Cancer Genome Consortium (ICGC)	https://icgc.org	
The Cancer Genome Atlas (TCGA)	http://cancergenome.nih.gov/	
COSMIC	http://cancer.sanger.ac.uk/cancergenome/projects/cosmic/	(1)
GeneCards	http://www.genecards.org	(2)
NCI Pathway Interaction Database	http://pid.nci.nih.gov	(3)
**Drugs and therapeutics**		
Drug Bank	http://www.drugbank.ca	(4)
Human Metabolome Database	http://www.hmdb.ca	(5)
Pubchem Compound	http://www.ncbi.nlm.nih.gov/pccompound/	(6, 7)
STITCH	http://stitch.embl.de/	(8)
Food and Drug Administration drug database	http://www.accessdata.fda.gov/scripts/cder/drugsatfda/index.cfm	
**Clinical trials**		
ClinicalTrials.gov	http://clinicaltrials.gov	
**Gene-molecule context**		
Cancer Cell Line Encyclopedia	http://www.broadinstitute.org/ccle/home	(9)
Project Achilles	http://www.broadinstitute.org/achilles	(10)
The Connectivity Map	http://www.broadinstitute.org/cmap/	
**Drug repurposing**		
PROMISCUOUS	http://bioinformatics.charite.de/promiscuous/index.php	(11)

REFERENCES:

1. Forbes SA, Bindal N, Bamford S, Cole C, Kok CY, Beare D, et al. COSMIC: mining complete cancer genomes in the Catalogue of Somatic Mutations in Cancer. Nucleic acids research. 2011;39:D945-50.
2. Rebhan M, Chalifa-Caspi V, Prilusky J, Lancet D. GeneCards: integrating information about genes, proteins and diseases. Trends in genetics : TIG. 1997;13:163.
3. Schaefer CF, Anthony K, Krupa S, Buchoff J, Day M, Hannay T, et al. PID: the Pathway Interaction Database. Nucleic acids research. 2009;37:D674-9.
4. Wishart DS, Knox C, Guo AC, Shrivastava S, Hassanali M, Stothard P, et al. DrugBank: a comprehensive resource for in silico drug discovery and exploration. Nucleic acids research. 2006;34:D668-72.
5. Wishart DS, Jewison T, Guo AC, Wilson M, Knox C, Liu Y, et al. HMDB 3.0--The Human Metabolome Database in 2013. Nucleic acids research. 2013;41:D801-7.
6. Bolton E WY, Thiessen PA, Bryant SH. PubChem: Integrated Platform of Small Molecules and Biological Activities. Annual Reports in Computational Chemistry. Oxford, UK: Elsevier; 2008. p. 217-40.
7. Wang Y, Suzek T, Zhang J, Wang J, He S, Cheng T, et al. PubChem BioAssay: 2014 update. Nucleic acids research. 2014;42:D1075-82.
8. Kuhn M, von Mering C, Campillos M, Jensen LJ, Bork P. STITCH: interaction networks of chemicals and proteins. Nucleic acids research. 2008;36:D684-8.
9. Barretina J, Caponigro G, Stransky N, Venkatesan K, Margolin AA, Kim S, et al. The Cancer Cell Line Encyclopedia enables predictive modelling of anticancer drug sensitivity. Nature. 2012;483:603-7.
10. Cheung HW, Cowley GS, Weir BA, Boehm JS, Rusin S, Scott JA, et al. Systematic investigation of genetic vulnerabilities across cancer cell lines reveals lineage-specific dependencies in ovarian cancer. Proceedings of the National Academy of Sciences of the United States of America. 2011;108:12372-7.
11. von Eichborn J, Murgueitio MS, Dunkel M, Koerner S, Bourne PE, Preissner R. PROMISCUOUS: a database for network-based drug-repositioning. Nucleic acids research. 2011;39:D1060-6.
